# Enhanced Identification of Postoperative Infections among Outpatients

**DOI:** 10.3201/eid1011.040784

**Published:** 2004-11

**Authors:** Andrew L. Miner, Kenneth E. Sands, Deborah S. Yokoe, John Freedman, Kristin Thompson, James M. Livingston, Richard Platt

**Affiliations:** * Harvard Medical School, Boston, Massachusetts, USA;; † Harvard Pilgrim Health Care, Boston, Massachusetts, USA;; ‡ Brigham and Women's Hospital, Boston, Massachusetts, USA;; § Beth Israel Deaconess Medical Center, Boston, Massachusetts, USA;; # Tufts Health Plan, Watertown, Massachusetts, USA

**Keywords:** cesarean section, puerperal infection, mastectomy, surgical wound infection, health maintenance organizations, ambulatory care, population surveillance, medical records, medical records systems, infection control, research

## Abstract

We investigated using administrative claims data to identify surgical site infections (SSI) after breast surgery and cesarean section. Postoperative diagnosis codes, procedure codes, and pharmacy information were automatically scanned and used to identify claims suggestive of SSI ("indicators") among 426 (22%) of 1,943 breast procedures and 474 (10%) of 4,859 cesarean sections. For 104 breast procedures with indicators explained in available medical records, SSI were confirmed for 37%, and some infection criteria were present for another 27%. Among 204 cesarean sections, SSI were confirmed for 40%, and some criteria were met for 27%. The extrapolated infection rates of 2.8% for breast procedures and 3.1% for cesarean section were similar to those reported by the National Nosocomial Infection Surveillance program but differ in representing predominantly outpatient infections. Claims data may complement other data sources for identification of surgical site infections following breast surgery and cesarean section.

## Abstract 2

Claims data complement other data sources for identification of surgical site infections following breast surgery and cesarean section.

The most commonly used methods for surgical site infection (SSI) surveillance are labor intensive, susceptible to variability, and relatively insensitive to SSI after hospital discharge (*1**–**17*). Automated diagnosis and treatment information created during routine healthcare delivery, if sufficiently accurate, could be used to improve SSI detection. Surveillance based on full-text electronic medical records has outperformed more widely used methods (*18**,**19*), but currently these records exist for a minority of procedures. Diagnosis, procedure, and pharmacy codes associated with insurance claims are widely available but provide less detailed information. Nevertheless, claims data after coronary artery bypass grafting (CABG) identified 45% more SSI than did traditional surveillance (*20*). Claims data also allowed comparison of infection rates between hospitals (*21*). We investigated the utility of claims data after breast surgery and cesarean section for infection surveillance; these procedures are among the most commonly performed. Assessing different procedure types is important because differences in the duration of hospitalization, inpatient management, postdischarge care, and practices in billing and reimbursement that underlie claims data may vary substantially among different types of procedures.

## Methods

### Breast Surgery

#### Automated Data

The study population was drawn from three different administrative claims systems within Harvard Pilgrim Health Care from July 1997 through February 1999 and one system of Tufts Health Plan from January 1996 through February 1999. All members had benefits that would be expected to generate inpatient and outpatient diagnosis and procedure claims; 90% of members also had pharmacy benefits (unpub. data). Breast procedures were identified by International Classification of Diseases 9th Revision Clinical Modification (ICD-9-CM) or Current Procedural Terminology (CPT) procedure codes (Appendix 1). Breast surgeries were divided into the following four categories on the basis of expected infection risk: 1) limited procedures, including reduction mammoplasty, mastopexy without implant, and mastectomy without axillary dissection or reconstruction; 2) procedures that involve implants; 3) mastectomy with axillary dissection; and 4) procedures that include reconstruction. Breast biopsies and local excisions were not studied. The unit of analysis was procedure, and members could contribute more than one. However, procedures were excluded if another qualifying breast surgery occurred during the preceding or subsequent 60 days.

We searched claims and pharmacy data during the 60 days after surgery for previously published diagnosis codes, procedure codes, and antimicrobial agent dispensing suggestive of infection (*21*). Six categories of "SSI indicators" included diagnosis codes associated with inpatient, emergency department and outpatient settings, procedure codes for wound care in any setting, procedure codes for wound culture in any setting, and antimicrobial agents. The data available about antimicrobial drugs were limited to the outpatient setting. We then applied an algorithm that estimated the probability of infection on the basis of the presence or absence of SSI indicators in the six categories (*21**,**22*). The probability is derived from a logistic regression equation that assigns weights for each of the SSI indicator types for the individual patient. This probability could range from 0.006 for procedures with no SSI indicators to 0.998 for procedures with indicators of all six types.

#### Record Review

We reviewed records from all procedures with a predicted probability of infection >0.03. These constituted 96% of procedures with any SSI indicator. We obtained records from as many of the following as could be identified: the surgeon who billed for the initial procedure, the patient's primary care provider at the time of surgery, and full-text electronic ambulatory records (one claims system). For procedures with an indicator from a hospital or emergency department, we also contacted the institution that submitted the first such claim. From outpatient providers, we requested all notes during the 60 days after surgery, and from hospitals and emergency departments, we requested a discharge summary or progress notes. Initial requests were mailed, and providers who did not respond were telephoned 3–6 weeks later.

Full-text medical records were reviewed in two stages. A primary reviewer recorded the signs and symptoms during the 60 days after surgery that make up the National Nosocomial Infection Surveillance (NNIS) system definitions for SSI (*4*). If any signs or symptoms were found, an infectious disease physician experienced in clinical research performed a secondary review and classified the record as follows: 1) no signs or symptoms present, 2) some signs or symptoms of infection present without meeting the full NNIS definition, or 3) NNIS definition satisfied. The secondary reviewer also recorded the depth of SSI, if evident in the medical record. Discrepancies between primary and secondary reviews were resolved by two reviewers. The primary reviewer also determined whether or not the received records were adequate for inclusion in further analysis. Outpatient records were considered adequate if the record had notes for the 6 weeks after surgery, regardless of whether they contained specific reference to postoperative care or provided any explanation for the indicator that prompted the review. Hospital records were considered adequate if they contained notes from the identified admission or emergency department visit.

#### Completeness of Data

We compared the number of ambulatory claims, diagnoses associated with these claims, prescriptions before and after delivery, each SSI indicator type, and SSI confirmation rate among the four claims systems for each 6-month interval. The overall rate of SSI indicators and the confirmation rate for adequate records were not different. Small, but statistically significant, differences were noted among claims systems in patient age and the number of diagnoses on days with ambulatory claims. In one claim system, procedure codes for wound care were found after 5% of surgeries. This indicator type was associated with <1% of surgeries in the other three claims systems. The rates of procedure codes for wound culture and inpatient diagnosis codes were slightly different. A 10% drop over time occurred in the number of procedures with ambulatory claims in two systems, but this drop was not associated with a change in the rate of ambulatory diagnosis SSI indicators.

#### Analysis

We used the χ^2^ test to compare categorical values and the Kruskal-Wallis tests for continuous variables. Analyses were performed with SAS (SAS, Cary, NC) for Unix version 8.2. We extrapolated the full SSI rate by multiplying the rate of confirmed infection among adequate charts by the proportion of procedures with a predicted probability of infection >0.03. We were prepared to compare infection rates among hospitals, but too few had a sufficiently high volume.

### Cesarean Section

#### Automated Data

This study population comprised patients with ICD-9-CM procedure codes for cesarean section (Appendix 1) and was limited to the three administrative claims systems at Harvard Pilgrim. Additional exclusion criteria were age <16 years or >50 years and sex recorded as male. Records were searched for 30 days postoperatively rather than 60 days, and the SSI indicator list for cesarean section differed from that for breast procedures (Appendix 2). These codes were chosen to identify SSI, including endometritis but not mastitis or urinary tract infection. We ignored SSI indicators associated with procedures having a diagnosis code suggestive of mastitis (mastitis indicators) (Appendix 3).

#### Record Review

We obtained records for procedures with an SSI or mastitis indicator, as described for breast procedures. For cesarean sections we requested records from all of the following that were applicable and available through claims: any obstetricians who performed the cesarean section, submitted the first outpatient claim with an SSI indicator, or was associated with most prenatal visits; the first hospital or emergency room that generated an SSI indicator; and full-text electronic ambulatory records (one claims system). Of received charts, the greatest portion (44%) came from the delivering obstetrician.

In addition to identifying SSI, the primary and secondary reviewers also assessed the presence of endometritis and mastitis by using the NNIS definitions (*4*). Only events occurring during the first 30 postoperative days were considered. Reliability between raters was assessed for the primary review (κ = 0.86 for identification of any sign or symptom, κ = 0.62 for identifying adequate charts).

#### Completeness of Data

We performed the same comparisons among claims systems for each 6-month period as was done for breast procedures. Differences occurred in patient age, number of prescriptions before and after surgery, and days with ambulatory claims. The differences in SSI indicators were less pronounced than those noted for breast procedures. The 10% decrease in ambulatory care claims over time was found for cesarean sections as well.

### Analysis

In addition to the analyses described for breast procedures, we compared SSI rates among institutions with >150 procedures. We used logistic regression analysis to compare the proportions of cesarean sections with an SSI indicator at each hospital, adjusting for age (tertiles), secular trend (6-month intervals), and claims system. An interaction term "system*hospital" was tested to determine whether including data from multiple claims systems was appropriate when comparing hospitals' rates of SSI indicators.

## Results

### Breast Surgery

A total of 1,943 breast procedures were eligible (86% of all procedures identified). Most procedures had associated postoperative prescribing and ambulatory claims (Table 1). The most common SSI indicators were antimicrobial drug dispensing and ambulatory diagnosis codes; 22% of procedures had at least one indicator.

**Table 1:** Breast surgeries and cesarean sections identifiable from claims data^a^

Characteristics	Breast procedures	Cesarean section
No. of procedures	1,943	4,859
Median patient age in y (interquartile range)	48 (39–55)	32 (28–35)
% with prescriptions within 30 days after surgery	62	61
% with prescriptions in the 6 months before surgery	66	23
Postoperative days w/ambulatory claims^b^	6 (2–11)	1 (0–2)
Diagnoses on days w/ambulatory claims^b^	3 (2–4)	1 (1–2)
SSI indicator categories^c^		
Inpatient diagnosis (%)	30 (1.5)	63 (1.3)
Principally outpatient indicators (%)		
Ambulatory setting diagnosis (excludes ED)	173 (8.9)	112 (2.3)
Antimicrobial drugs in ambulatory setting	279 (14)	277 (5.7)
Wound culture	20 (1.0)	124 (2.6)
Wound care	33 (1.7)	11 (0.2)
Emergency department diagnosis	25 (1.3)	25 (0.5)
Any SSI indicator (%)	426 (22)	474 (10)

^a^SSI, surgical site infection, ED, emergency department. ^b^Postoperative days 0–60 for breast surgeries, 0–30 for cesarean section. ^c^Number of procedures (percent of total) with at least one SSI indicator of the listed type in the 60 days (breast procedures) or 30 days (cesarean sections) after surgery.

We requested records for 395 procedures (96% of those with an indicator) and received adequate documentation for 209 (53%) (Table 2). An infection was confirmed by NNIS criteria for 38 (18%); 28 (13%) had signs or symptoms that suggested infection without meeting the criteria. Among the 104 with records that included an explanation for the SSI indicator, 37% had a confirmed SSI, and 27% had signs or symptoms. Twenty (53%) confirmed infections were superficial; 12 (32%) were deep or in an organ space, and the depth could not be determined for 6 infections. Other infections or noninfectious causes explained the infection indicator for a minority of procedures, but in 50% of cases, neither the indicator nor a likely cause was mentioned.

**Table 2:** Results of medical record review^a^

Characteristic	Breast (%)	Cesarean (%)
Procedures with possible SSI^b^	410 (21)	474 (10)
Requested 1 or more records (% of those with possible SSI)^c^	395 (96)	443 (93)
Records received (% of requested)	295 (75)	342 (77)
Adequate record received (% of requested)	209 (53)	255 (58)
No. among adequate records (% of adequate records)^d^		
Confirmed SSI	38 (18)	82 (32)
Some signs and symptoms of SSI, does not meet criteria	28 (13)	56 (22)
No evidence of SSI		
Another infection found, responsible for indicator	9 (4)	38 (15)
SSI indicator explained, not caused by infection	29 (14)	28 (11)
SSI indicator could not be explained	105 (50)	51 (20)

^a^SSI, surgical site infection. ^b^For breast procedures, those with possible SSI are procedures with predicted probability of infection >0.03. For cesarean section, procedures with possible SSI are those with any SSI indicator and no mastitis indicators. ^c^Common reasons for not requesting records were the following: member's information restricted, no provider could be identified from claims, or no current contact information could be obtained for a provider. ^d^Breast procedure outcomes based on 60 postoperative days; cesarean section outcomes based on 30 postoperative days.

Of the 38 infections we identified, 28 (74%) were identified during the first 30 days, which yielded an extrapolated infection rate based on NNIS (30-day) criteria of 2.8%. SSI indicators were found during a hospital admission for 40 (2.1%) of the 1,943 procedures, and SSI was confirmed for 20%, which yielded an inpatient extrapolated SSI rate of 0.4%. The similarly calculated outpatient SSI rate was 2.4%. Over the full 60 days reviewed, the infection rate was 3.8%.

**Figure 1:**
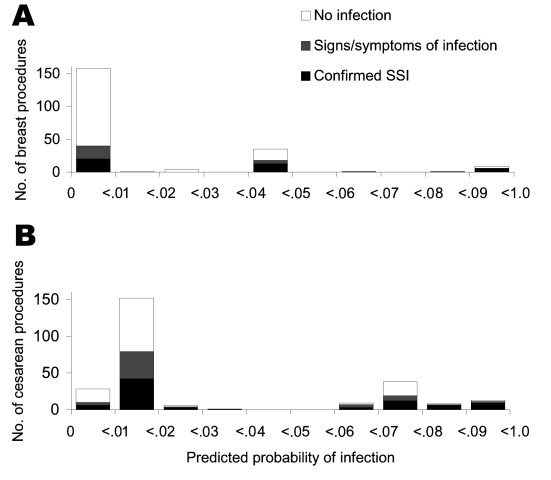
Infectious outcomes by predicted probability of surgical site infections (SSI) calculated from SSI indicators for A) breast procedures and B) cesarean sections. Shown are all procedures with adequate documentation, which excludes 80%–90% of procedures with no SSI indicator and predicted probability of infection at baseline, 0.006. Predicted probability of infection is based on the categories of SSI indicators found in claims and pharmacy records. The infectious outcomes for breast procedures are based on postoperative days 0–60; cesarean section outcomes are from days 0–30.

The confirmation rate for patients with SSI indicators increased with the predicted probability of infection (Figure 1A), from 13% for those with a predicted probability <0.1 (76% of procedures with indicators) to 37% (13/35) for procedures with predicted probabilities of 0.4 to 0.5, and 50% for the 10 procedures with a predicted probability >0.8.

**Figure 2:**
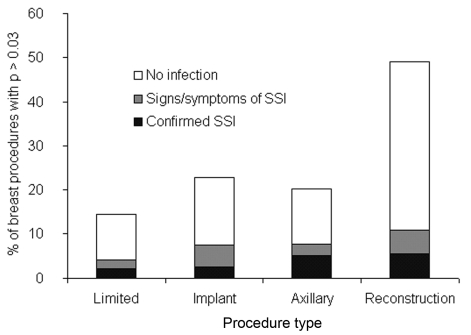
Infectious outcomes among four categories of breast procedure. Each bar represents all procedures with predicted probability of infection >0.03. Shown are 60-day outcomes extrapolated from the rates among procedures with adequate records. p, predicted probability of infection; SSI, surgical site infection; limited, reduction mammoplasty, mastopexy without implant, and mastectomy without axillary dissection or reconstruction; implant, breast procedures with an implant; axillary, breast procedures with axillary dissection; reconstruction, breast procedures with reconstruction.

Among the four types of breast surgery, the occurrence of infection indicators ranged from 16% among limited procedures to 50% among procedures with reconstruction (Figure 2). The infection indicator type most responsible for this difference was antimicrobial agents, which were found after 41% of procedures with reconstruction but only 9% of limited procedures. The extrapolated 60-day infection rates among the four surgery types was 2.2% for limited procedures, 2.5% among procedures with implants, 5.2% among surgeries involving axillary dissection, and 5.5% among surgeries with reconstruction. Not enough hospitals had >100 procedures to allow comparisons.

### Cesarean Section

A total of 4,859 (98% of those identified) cesarean sections were eligible. Antimicrobial drug prescribing was the most common SSI indicator, and 10% of deliveries had an indicator of some type (Table 1). One or more requests could be made for 443 (93%) cesarean sections, and adequate records were received for 255 (58%) (Table 2). SSI were confirmed more often than for breast procedures: 82 deliveries (32% of those with adequate records) had a confirmed SSI, and another 56 (22%) had signs or symptoms. Among the 204 with records that included an explanation for the SSI indicator, 40% had a confirmed SSI, and 27% had signs or symptoms. Among confirmed SSI, 45% were superficial incisional, 6% were deep incisional, 24% were endometritis, and depth could not be determined for 24%. The extrapolated inpatient infection rate of 0.6% and the outpatient rate of 2.5% combine for an overall 3.1% 30-day SSI rate.

The distribution of predicted probability of infection among procedures with SSI indicators differed from that for breast procedures in having two discrete peaks (Figure 1B). Among the 73% of adequately documented procedures with predicted probability <0.4, the SSI confirmation rate was 28%. Above predicted probability 0.6 the confirmation rate was 44% (30/68).

**Figure 3:**
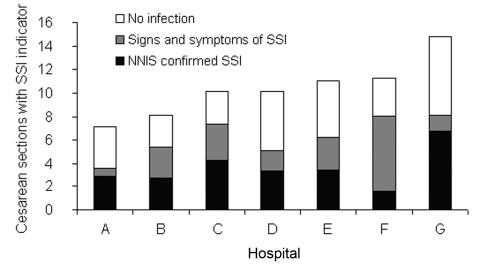
Hospital-specific infectious outcomes among cesarean sections with surgical site infection (SSI) indicators. Each bar represents all deliveries with potential SSI (n = 10–50), with outcomes extrapolated from those for whom adequate records were returned. Below each bar is the odds ratio (95% confidence interval) for a delivery having an SSI indicator at each hospital, adjusted for age, claims system, and 6-month interval. Hospital A (reference), hospital B (OR 1.2 [95% CI 0.7–2.1]), hospital C (OR 1.7 [95% CI 1.0–2.9]), hospital D (OR 1.8 [95% CI 1.1–2.9]), hospital E (OR 1.7 [95% CI 1.0–2.8], hospital F (OR 1.7 [95% CI 0.9–3.1], hospital G (OR 2.3 [95% CI 1.3–4.0). NNIS, National Nosocomial Infections Surveillance; CI, confidence interval.

Seven hospitals performed 150 or more cesarean sections. The proportion of each hospital's cesarean sections with an SSI indicator was 7.2%–14.8%, with confirmation rates that extrapolated to overall SSI rates of 1.6% to 6.7% (Figure 3). The hospitals' overall rates of confirmed SSI or signs and symptoms of SSI correlated with their rates of SSI indicators (p = 0.03). Three hospitals had an SSI indicator rate that was significantly greater than that of the hospital with the lowest SSI rate (hospital A in Figure 3), after adjusting for patients' age, claims system, and 6-month interval. We found no evidence of significant differences between claims systems in ranking hospitals.

Mastitis indicators were found after 22 deliveries, 15 of which also had an SSI indicator that would have identified them as "potential SSI" had they not been specifically excluded. Among the 14 for which an adequate record was obtained, 6 (43%) cases met the NNIS criteria for mastitis, and 5 (36%) had signs or symptoms of mastitis. None had a confirmed SSI.

## Discussion

These findings support the major conclusion of earlier work with CABG procedures (*20*): claims data may be a useful adjunct to conventional surveillance for SSI. The strength of the claims data for breast procedures and cesarean delivery was in identifying SSI treated in the ambulatory setting, with >80% identified solely through ambulatory claims. In contrast, only 16% of SSI identified by NNIS occurred in the ambulatory setting (*9*). We believe claims data did not identify many of the SSI that occurred among inpatients because our overall extrapolated SSI rates were approximately equal to the rates published by NNIS during the period of this study. For breast procedures, the NNIS rate during the decade that included our study period was 2.1% (*23*), compared to our extrapolated rate of 2.8%. We note that the NNIS definition of breast procedure includes four less extensive procedures, including open biopsy and lumpectomy, that we did not study (*24*). For cesarean section, the 3.1% overall SSI rate identified by claims was almost identical to the 3.2% identified by NNIS for essentially the same procedures (*23**,**24*). The finding that claims data were apparently more useful for identifying postdischarge SSI than inpatient SSI is a contrast to our finding in CABG procedures, that claims data appeared to identify SSI occurring in both inpatient and outpatient settings. The relative performance of claims data and routine inpatient surveillance would best be addressed by comparing results in the same institutions during comparable periods.

The overall rates at which SSI indicators identified true SSI were comparable to those we previously described for CABG procedures (*21*), if one applies the same criteria, considering only records that provided some explanation of the claims-based indicator (proportion with confirmed SSI or signs and symptoms: 63% for breast surgery, 68% for cesarean sections, 66% for CABG). The proportions of breast surgery and cesarean section patients whose records fully satisfied criteria for SSI were somewhat lower (37% and 40%) than was the case for our CABG population, for whom 53% of procedures with any indicator had a confirmed SSI (*21*). We believe these findings represent minimum estimates of the predictive value of the SSI indicators and of the extrapolated infection rates because many of the medical records we received did not identify the reason for the claim that yielded the SSI indicator or because the description of an abnormal surgical site contained too little detail to confirm an infection that may have been present. For CABG, the lower rates of procedures with signs and symptoms that did not fulfill all SSI criteria may have been attributable to more thorough documentation in the ambulatory medical records after CABG procedures.

The frequent dispensing of antistaphylococcal antimicrobial agents during the month after discharge, especially after 14% of breast procedures, bears consideration beyond its effect on lowering the predictive value of this SSI indicator. Much of this dispensing may have been for extended perioperative prophylaxis, a practice at variance with the Joint Commission for Accreditation of Healthcare Organizations' recent guideline limiting postoperative antimicrobial prophylaxis to a single day (*25*). As noted above, some of these antimicrobial courses may have been prescribed as treatment for diagnosed bacterial infections for which the documentation did not satisfy NNIS criteria or for presumed bacterial infections. Some courses may have been a prophylaxis regimen that would be considered inappropriate by current standards. Whatever the reasons, additional attention to postoperative antimicrobial drug use will be worthwhile, since if this use continues to be common, it may represent a large amount of currently undocumented illness or inappropriate antimicrobial drug use.

The predictive value of SSI indicators after cesarean section was reduced by the relatively common occurrence of infections at sites other than the surgical incision. Thus, these indicators may be useful in detecting postoperative infectious illness other than SSI. Also, for both breast procedures and cesarean sections, and in contrast to our experience with CABG procedures, the patients with an SSI indicator could be partitioned into groups with higher or lower likelihood of confirmed SSI.

We have no direct information about the status of approximately one quarter of patients with SSI indicators for whom no medical records could be obtained. Although we did not collect information systematically about missing records, most were likely missing for reasons unrelated to their clinical status, e.g., because the clinicians could not be contacted, the patients' records were no longer available, or because of the refusals of some institutions to provide records. While these missing patients may have had higher infection rates than the ones whose records we were able to review, we observed no important difference in the extrapolated infection rates between patients in one of the systems for which we obtained all requested ambulatory records because it used an electronic medical record system.

These results affirm the ability to combine data from multiple systems, which may be necessary to obtain enough information to estimate hospital-specific rates. The claims data for breast procedures from two health plans and the three administrative systems within one of those organizations were comparable in the proportion of procedures with most of the types of SSI indicators and in the rate at which identified procedures were confirmed to have an SSI. The higher rate of procedure codes for wound care in one data system probably represented a difference in coding practice or data structure. Claims systems do not need identical SSI indicator rates or confirmation rates for their data to be pooled, as long as this difference is controlled for when making other comparisons. Understanding whether a particular claims system is suitable for surveillance is important. For instance, if surgeons are paid a fixed price for a procedure and all postoperative care, then the claims are unlikely to provide indicators for ambulatory care. Similarly, antimicrobial indicators are much less meaningful if patients do not have a drug benefit or if the claims are "carved out," i.e., paid by another organization. Finally, for all data systems, routine checks should be performed for completeness, consistency, and accuracy of the data.

These claims-based indicators are not synonymous with infection and should not be used by themselves to categorize hospitals or practice groups as having high rates of complications. Instead, if additional evaluation supports the usefulness of claims data for this purpose, then these data might be used to identify a limited number of hospitals that merit additional follow-up to determine whether their rates of SSI are unusually high. The three hospitals with higher rates of SSI indicators after cesarean section included the two with the highest extrapolated confirmed SSI rates, which suggests that focusing resources on understanding whether any of these three hospitals had increased rates because of remediable factors may have been effective. Valid reasons may exist for institutions' confirmed SSI rates to differ; for instance case-mix might differ. Additionally, any investigation of a specific hospital's indicator rate should begin by determining whether these elevated rates result from differences in the way claims for its patients are prepared or processed.

Widely available claims data, like those used here, may form the basis of an efficient system for identifying patients with increased likelihood of having had an SSI after breast surgery and cesarean section, as has been reported for CABG. If these results are confirmed, then assessing claims may be a useful adjunct to other forms of surveillance and might replace other methods for postdischarge surveillance.

## Appendix 1

### ICD-9-CM and CPT procedure codes used to identify surgeries of interest

#### Breast Surgeries

##### Category 1: Limited procedures (reduction mammoplasty, mastopexy without implant, and mastectomy without axillary dissection or reconstruction)

##### ICD-9-CM procedure codes

85.23 Subtotal mastectomy

85.31 Unilateral reduction mammoplasty

85.32 Bilateral reduction mammoplasty

85.34 Other unilateral subcutaneous mammectomy

85.36 Other bilateral subcutaneous mammectomy

85.4 Mastectomy

85.41 Unilateral simple mastectomy

85.42 Bilateral simple mastectomy

85.45 Unilateral radical mastectomy

85.46 Bilateral radical mastectomy

85.50 Augmentation mammoplasty, not otherwise specified

85.6 Mastopexy

##### CPT procedure codes

19140 Mastectomy for gynecomastia

19160 Mastectomy, partial

19180 Mastectomy, simple

19182 Mastectomy, subcutaneous

19316 Mastopexy

19318 Reduction mammaplasty

19324 Mammaplasty augmentation, without prosthetic implant

##### Category 2: Procedures that involve implants

##### ICD-9-CM procedure codes

85.33 Unilateral subcutaneous mammectomy with synchronous implant

85.35 Bilateral subcutaneous mammectomy with synchronous implant

85.53 Unilateral breast implant

85.54 Bilateral breast implant

##### CPT procedure codes

19325 Mammaplasty, augmentation, with prosthetic implant

19340 Immediate insertion of breast prosthesis following mastopexy or mastectomy

19342 Delay insertion of breast prosthesis following mastopexy or mastectomy

19370 Open periprosthetic capsulotomy breast

19371 Periprosthetic capsulectomy breast

##### Category 3: Procedures that involve axillary dissection

ICD-9-CM procedure codes

85.43 Unilateral extended simple mastectomy

85.44 Bilateral extended simple mastectomy

85.47 Unilateral extended radical mastectomy

85.48 Bilateral extended radical mastectomy

##### CPT procedure codes

19162 Mastectomy partial, with axillary lymphadenectomy

19200 Mastectomy, radical, including pectoral muscles and axillary lymph nodes

19220 Mastectomy, radical, including pectoral muscles, and axillary and internal mammary lymph nodes

19240 Mastectomy, modified radical, including axillary lymph nodes, with or without pectoralis minor muscle, but excluding pectoralis major muscle

##### Category 4: Involve reconstruction and/or flaps

##### ICD-9-CM procedure codes

85.7 Total reconstruction of breast

##### CPT procedure codes

19357 Breast reconstruction, immediate or delayed, with tissue expander, including subsequent expansion

19361 Breast reconstruction with latissimus dorsi flap, with or without prosthetic implant

19364 Breast reconstruction with free flap

19366 Breast reconstruction with other technique

19367 Breast reconstruction with transverse rectus abdominis myocutaneous flap (TRAM), single pedicle, including closure of donor site

19369 Breast reconstruction with transverse rectus abdominis myocutaneous flap (TRAM), double pedicle, including closure of donor site

##### Cesarean Section

##### ICD-9-CM procedure codes

74.0 Classical cesarean section

74.1 Low cervical cesarean section

74.2 Extraperitoneal cesarean section

74.4 Cesarean section of other specified type

74.9 Cesarean section of unspecified type

74.99 Other cesarean section of unspecified type

## Appendix 2

### Diagnosis codes, procedure codes, and antibiotics that constitute SSI indicators for Cesarean section

#### ICD-9-CM Diagnosis codes (used in hospital, emergency department, or outpatient settings)

038 Septicemia

038.0 Streptococcal septicemia

038.1 Staphylococcal septicemia

038.10 Unspecified staphylococcal septicemia

038.11 Staphylococcus aureus septicemia

038.19 Other staphylococcal septicemia

038.3 Septicemia due to anaerobes

038.4 Septicemia due to other gram-negative organisms

038.40 Septicemia due to unspecified gram-negative organism

038.42 Septicemia due to Escherichia coli (E. coli)

038.43 Septicemia due to pseudomonas

038.44 Septicemia due to serratia

038.49 Other septicemia due to gram-negative organism

038.8 Other specified septicemia

038.9 Unspecified septicemia

040.0 Gas gangrene

040.8 Other specified bacterial diseases

040.82 Toxic shock syndrome

040.89 Other specified bacterial diseases

041 Bacterial infection in conditions classified elsewhere and of unspecified site

041.0 Streptococcus infection in conditions classified elsewhere and of unspecified site

041.00 Unspecified streptococcus infection in conditions classified elsewhere and of unspecified site

041.01 Streptococcus infection in conditions classified elsewhere and of unspecified site, group A

041.03 Streptococcus infection in conditions classified elsewhere and of unspecified site, group C

041.04 Streptococcus infection in conditions classified elsewhere and of unspecified site, group D

041.05 Streptococcus infection in conditions classified elsewhere and of unspecified site, group G

041.09 Other streptococcus infection in conditions classified elsewhere and of unspecified site

041.1 Staphylococcus

041.10 Staphylococcus, unspecified

041.11 Staphylococcus aureus

041.19 Other Staphylococcus

041.3 Friedlander's bacillus infection in conditions classified elsewhere and of unspecified site

041.4 Escherichia coli (E. coli) infection in conditions classified elsewhere, unspecified site

041.6 Proteus (mirabilis) (morganii) infection, classified elsewhere and of unspecified site

041.7 Pseudomonas infection in conditions classified elsewhere and of unspecified site

041.8 Other specified bacterial infections in conditions classified elsewhere and of unspecified site

041.82 Bacillus fragilis infection in conditions classified elsewhere and of unspecified site

041.83 Clostridium perfringens infection in conditions classified elsewhere and of unspecified site

041.84 Infection due to other anaerobes in conditions classified elsewhere and of unspecified site

041.85 Infection due to other gram-negatives, conditions classified elsewhere and of unspecified site

041.89 Infection due to other specified bacteria in conditions classified elsewhere, unspecified site

041.9 Bacterial infection, unspecified, in conditions classified elsewhere and of unspecified site

614.0 Acute salpingitis and oophoritis

614.2 Salpingitis and oophoritis not specified as acute, subacute, or chronic

614.3 Acute parametritis and pelvic cellulitis

614.5 Acute or unspecified pelvic peritonitis, female

614.9 Unspecified inflammatory disease of female pelvic organs and tissues

615 Inflammatory diseases of uterus, except cervix

615.0 Acute inflammatory disease of uterus, except cervix

615.9 Unspecified inflammatory disease of uterus

670 Major puerperal infection

670.0 Major puerperal infection

670.00 Major puerperal infection, unspecified as to episode of care

670.02 Major puerperal infection, delivered, with mention of postpartum complication

670.04 Major puerperal infection, postpartum

672 Pyrexia of unknown origin during the puerperium

672.0 Pyrexia of unknown origin during the puerperium

672.00 Puerperal pyrexia of unknown origin, unspecified as to episode of care

672.02 Puerperal pyrexia of unknown origin, delivered, with mention of postpartum complication

672.04 Puerperal pyrexia of unknown origin, postpartum

673.3 Obstetrical pyemic and septic embolism

673.30 Obstetrical pyemic and septic embolism, unspecified as to episode of care

673.31 Obstetrical pyemic and septic embolism, with delivery, with or without mention of antepartum condition

673.32 Obstetrical pyemic and septic embolism, with delivery, with mention of postpartum complicaton

673.33 Obstetrical pyemic and septic embolism, antepartum

673.34 Obstetrical pyemic and septic embolism, postpartum

682 Other cellulitis and abcess

682.2 Cellulitis and abscess of trunk

682.5 Cellulitis and abscess of buttock

686 Other local infection of skin and subcutaneous tissue

686.8 Other specified local infections of skin and subcutaneous tissue

686.9 Unspecified local infection of skin and subcutaneous tissue

780.6 Fever of unknown origin

790.7 Bacteremia

996.6 Infection and inflammatory reaction due to internal prosthetic device, implant, and graft

996.60 Infection and inflammatory reaction due to unspecified device, implant, and graft

996.62 Infection and inflammatory reaction due to other vascular device, implant, and graft

996.69 Infection and inflammatory reaction due to other internal prosthetic device, implant, and graft

998.5 Postoperative infection, not elsewhere classified

998.51 Infected postoperative seroma

998.59 Other postoperative infection

### Wound culture codes (outpatient setting)

#### ICD-9-CM

90.5# - Microscopic examination of blood

1 bacterial smear

2 culture

3 culture and sensitivity

### Current Procedural Terminology (CPT)

87040 Culture bacterial; blood with isolation and presumptive identification of isolates

87070 Culture bacterial; any source except urine, blood or stool, with isolation and presumptive identification of isolates

87071 Culture bacterial; quantitative, aerobic with isolation and presumptive identification of isolates, any source except urine, blood or stool

87072 Culture bacterial; any source by kit with isolation and identification

87073 Culture bacterial; quantitative, anaerobic with isolation and presumptive identification of isolates, any source except urine, blood, or stool

87075 Culture bacterial; any source, anaerobic and presumptive identification of isolates

87076 Culture bacterial; anaerobic isolate additional methods required for definitive identification, each isolate

87077 Culture bacterial; aerobic isolate additional methods required for definitive identification, each isolate

87081 Culture, presumptive, pathogenic organisms, screening only

87082 Culture of specimen by kit

87083 Culture of specimen by kit

87084 Culture, presumptive, pathogenic organisms, screening only; with colony estimation from density chart

87085 Culture, bacterial; quantitative colony count, urine

### Wound Care Codes

#### ICD-9-CM

86.01 Aspiration of skin and subcutaneous tissue (abscess, hematoma, or seroma)

86.04 Other incision with drainage of skin and subcutaneous tissue

86.22 Excisional debridement of wound, infection, or burn

#### CPT

10060 Incision and drainage of abscess; simple or single

10061 Incision and drainage of abscess; complicated or multiple

10160 Puncture aspiration of abscess, hematoma, bulla, or cyst

10180 Incision and drainage, complex, postoperative wound infection

11000 Debridement of extensive eczematous or infected skin; up to 10% of body surface

11001 Debridement of extensive eczematous or infected skin; additional 10% of body surface

### Antibiotics

Amoxicillin

Amoxicillin/Clavulanate

Ampicillin

Cefaclor

Cefadroxil

Cefixime

Cefprozil

Cefuroxime

Cephalexin

Cephradine

Ciprofloxacin

Clindamycin

Dicloxacillin

Doxycycline

Levofloxacin

Metronidazol

Minocycline

Ofloxacin

Oxacillin

Penicillin VK

Vancomycin

## Appendix 3

### Diagnosis codes that constitute mastitis indicators for cesarean section

#### ICD-9-CM Diagnosis codes (used in hospital, emergency department, or outpatient settings)

675 Infection of the breast and nipple associated with childbirth

675.0 Infection of nipple associated with childbirth

675.00 Infection of nipple associated with childbirth, unspecified as to episode of care

675.01 Infection of nipple associated with childbirth, delivered, with or without mention of antepartum condition

675.02 Infection of nipple associated with childbirth, delivered with mention of postpartum complication

675.03 Infection of nipple, antepartum

675.04 Infection of nipple, postpartum

675.1 Abscess of breast associated with childbirth

675.10 Abscess of breast associated with childbirth, unspecified as to episode of care

675.11 Abscess of breast associated with childbirth, delivered, with or without mention of antepartum condition

675.12 Abscess of breast associated with childbirth, delivered, with mention of postpartum complication

675.13 Abscess of breast, antepartum

675.14 Abscess of breast, postpartum

675.2 Nonpurulent mastitis associated with childbirth

675.20 Nonpurulent mastitis, unspecified as to episode of prenatal or postnatal care

675.21 Nonpurulent mastitis, delivered, with or without mention of antepartum condition

675.22 Nonpurulent mastitis, delivered, with mention of postpartum complication

675.23 Nonpurulent mastitis, antepartum

675.24 Nonpurulent mastitis, postpartum

675.8 Other specified infection of the breast and nipple associated with childbirth

675.80 Other specified infection of breast and nipple associated with childbirth, unspecified to episode

675.81 Other specified infection of the breast and nipple associated with childbirth, delivered, with or without mention of antepartum condition

675.82 Other specified infection of the breast and nipple associated with childbirth, delivered, with mention of postpartum complication

675.83 Other specified infection of the breast and nipple, antepartum

675.84 Other specified infection of the breast and nipple, postpartum

675.9 Unspecified infection of the breast and nipple associated with childbirth

675.90 Unspecified infection of the breast and nipple, unspecified as to prenatal or postnatal episode

675.91 Unspecified infection of the breast and nipple associated with childbirth, delivered, with or without mention of antepartum condition

675.92 Unspecified infection of the breast and nipple associated with childbirth, delivered, with mention of postpartum complication

675.93 Unspecified infection of the breast and nipple, antepartum

675.94 Unspecified infection of the breast and nipple, postpartum
